# Correlative multiple porosimetries for reservoir sandstones with adoption of a new reference-sample-guided computed-tomographic method

**DOI:** 10.1038/srep30250

**Published:** 2016-07-22

**Authors:** Jae Hwa Jin, Junho Kim, Jeong-Yil Lee, Young Min Oh

**Affiliations:** 1 Korea Institute of Geoscience and Mineral Resources, 124 Gwahak-ro, Yuseong-gu, Daejeon, 34132 Korea

## Abstract

One of the main interests in petroleum geology and reservoir engineering is to quantify the porosity of reservoir beds as accurately as possible. A variety of direct measurements, including methods of mercury intrusion, helium injection and petrographic image analysis, have been developed; however, their application frequently yields equivocal results because these methods are different in theoretical bases, means of measurement, and causes of measurement errors. Here, we present a set of porosities measured in Berea Sandstone samples by the multiple methods, in particular with adoption of a new method using computed tomography and reference samples. The multiple porosimetric data show a marked correlativeness among different methods, suggesting that these methods are compatible with each other. The new method of reference-sample-guided computed tomography is more effective than the previous methods when the accompanied merits such as experimental conveniences are taken into account.

Porosity in reservoir rocks is a yardstick for estimating the hydrocarbon reserve, i.e., the amount of oil and gas in place (OGIP). The traditional Dean Stark and Retort methods of early 1900s have been exploited so far to extract and quantify the matter of hydrocarbon in the rock sample which is also indicative of the reservoir porosity^1^,^2^,^3^. For more rapid, direct porosity measurements, researchers have developed different means of measurement such as mercury intrusion^4^,^5^ (MI), helium injection^3^ (HI), petrographic image analysis^6^,^7^ (PIA), and, more recently, computed-tomographic (CT) methods (e.g. refs 8, 9, 10, 11, 12). These methods have, however, yielded equivocal results, which are due to differences in principle and procedure between them. It is worth trying to minimize such discrepancies by means of further development or improvement of methods based on correct understanding of the reasons.

The method of mercury intrusion (MI) has been favored in that the unwetting nature of the liquid enables us to measure bulk volume (or bulk density) of samples at an ease^13^,^14^,^15^. It is also necessary to get matrix volume (or matrix density) for porosity calculation, which is somewhat straightforward in this method as the value is readable at the pressure condition of complete mercury saturation^4^. A porosimetric method using other liquids or gases could encounter difficulties in obtaining the bulk volume (or bulk density) of a rock sample, which can be adopted from a mercury method or other experiments^3^,^16^. When the bulk volume (bulk density) data are available, helium injection (HI) into the porous sample can be an efficient way to measure the pore volume because the helium molecule is small enough to infiltrate effectively into the micropores^4^,^17^.

The method of the petrographic image analysis (PIA) can settle the trouble of exacting the bulk volume measurement as it achieves the goal of porosity measurement by a direct calculation of individual areal portions of matrix and pores that can be binarized on an image slice^7^. This method, however, contains a strong cause of inaccurate porosity derivation in respect that the calculation is made on a single 2D image slice corresponding to the surface of a thin or polished section. Repeated manufacturing and observation of numerous 2D slices and statistic treatment of the data in multitude may help improve accuracy of porosimetric results, but may be time-consuming.

Utilization of computed tomography (CT) has an apparent merit in that it can produce an almost limitless number of tomogram slices for the image-based porosity determination^18^. Such slices enable us to segment the rock volume into numerous tiny voxels which can be classified into either side of matrix or pores. A similar function exists in the focused ion beam-scanning electron microscopy (FIB-SEM) system of better resolution (narrower slice gap) but the covered sample volume of the method is quite small thus debatable in respect of sample representativeness^19^. Although the CT method is evidently advantageous for the porosimetric measurement in volume, measurement accuracy depends on the resolution of individual voxels similarly to the case of pixels in the PIA method^20^. The resolution of CT is not adjustable as instantaneously as that of the PIA such that an appropriate resolution reflecting sample characteristics should be taken into account in the prior set up of CT components^12^.

In this paper, we attempt to test suitability of various porosimetric methods by comparing data derived from the methods applied sequentially to the two sets of Berea Sandstone samples which are practically the same with each other. Together with widely known methods, we have also adopted a new computed tomographic method using a reference material, as proposed by authors of this paper in an earlier literature^21^. Here we use one of Berea Sandstone (BS) samples as a reference in computed-tomographic scanning while the other one is scanned concurrently for the porosity measurement. We present multiple porosimetric data including those from the new reference-sample-guided computed tomography of which two different data sets were yielded under either low- or high-resolution control. With these data, we address how the new CT method is compatible with or even more effective than the other conventional porosimetric methods.

## Results

### General trend of multiple porosimetric data

**Figure 1:**
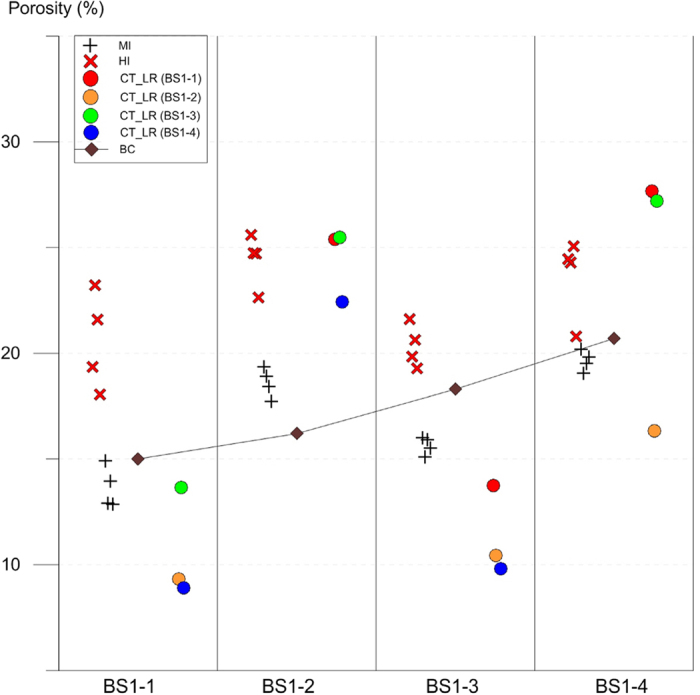
Results of multiple porosimetries with the methods of mercury intrusion (MI; plus), helium injection (HI; cross) and computed tomography of low resolution (CT_LR; circle, reference sample no. in parenthesis) that are plotted along with the arranged sample group numbers of the first sample set (See also supplementary Figs S1 and S2 for detailed sample information). Porosity data was also given (notified) by the company providing the samples, designated as BC porosities (diamonds connected with a line). Note a zigzagging trend of porosities in each method along with the arranged sample group numbers, i.e., porosities are low in BS1-1 and BS1-3, and high in BS1-2 and BS1-4. See also supplementary Table S4 for further detailed explanation of the CT_LR porosities.

**Figure 2:**
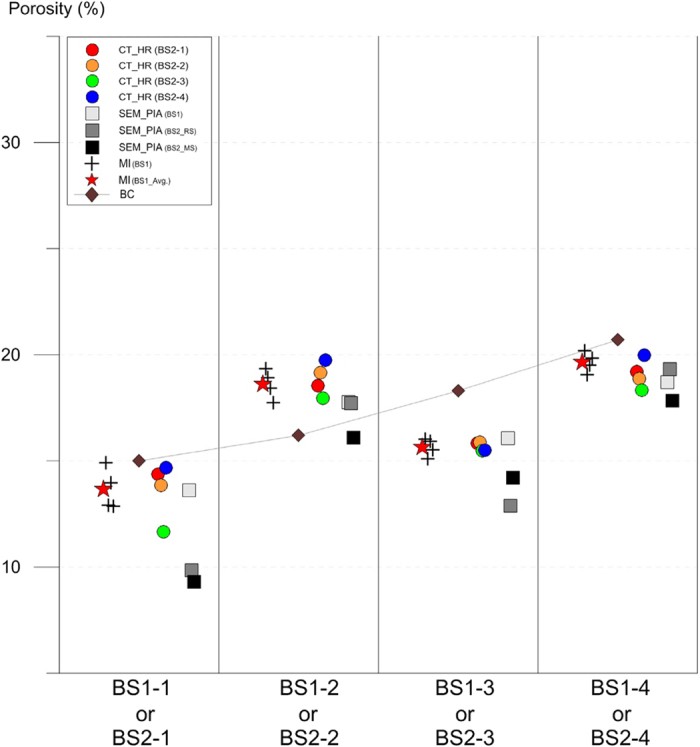
Results of multiple porosimetries that are plotted along with the arranged sample group numbers belonging to either the first or the second sample set (See also supplementary Figs S1, S2 and S3 for detailed sample information). Applied methods to the latter are computed tomography of high resolution (CT_HR; circle, reference sample no. in parenthesis) and petrographic image analysis on scanning electron microscopic images (SEM_PIA; square, further sample information in parenthesis). The porosities of mercury intrusion (MI; plus with star for average) method and Berea company notification (BC; diamond) are exactly the same as depicted in Fig. 1. Note a zigzagging trend of porosities along with the arranged sample group numbers, i.e., porosities are low in BS*-1 and BS*-3, and high in BS*-2 and BS*-4.

Figures 1 and 2 display results of multiple porosimetries applied to the two different sets of Berea Sandstone samples (BS1-* and BS2-*); each set comprises four sample groups, whereas each group consists of a number of mm-scale core plugs (See also supplementary Figs S1, S2 and S3). Each individual group in the first sample set has a “twin”, i.e., practically the same group in the second sample set. In Fig. 1, the results of helium injection (HI) and mercury intrusion (MI) methods as well as the low-resolution computed-tomographic (CT_LR) method are plotted along with the arranged sample group numbers of the first sample set. Porosities, notified by the company providing the samples, designated the Berea Sandstone company (BC) porosities, are also shown. In contrast, Fig. 2 shows the porosities of the high-resolution computed-tomographic (CT_HR) method applied to the second sample set. Also shown in Fig. 2 are porosities of the petrographic image analysis on the images of scanning electron microscopy, i.e., the SEM_PIA method, applied to either the first sample set or the second one.

Both Figs 1 and 2 indicate a conspicuous discrepancy between measured porosities and BC porosities. According to the notification of the Berea Sandstone company, the BC porosities increase almost linearly with arranged sample group numbers in each sample set, i.e., with the sample group numbers from BS1-1 to BS1-4 (Fig. 1) or from BS2-1 to BS2-4 (Fig. 2). However, in case of the commercial samples, a porosity value is determined at a small (cm-scale) part of a large (m-scale) block by a combination of various methods such as helium injection, mercury immersion and etc., and then the value is assigned to the whole block somewhat instantaneously, thus may not be proper to be used as testing purposes (personal e-mail communications; See also supplementary Information S1). According to our own multiple porosimetric data, a definite difference exists between the samples as well as the methods but clearly shown is a zigzag trend of the porosities, as recognized separately in each sample set by each method; porosities are low in the first and the third groups (nos. BS1-1 and BS1-3 in Fig. 1, and BS2-1 and BS2-3 in Fig. 2), whereas high in the second and the fourth groups (nos. BS1-2 and BS1-4 in Fig. 1, and BS2-2 and BS2-4 in Fig. 2). Moreover, the zigzag trends of both the first and second sample sets (Figs 1 and 2) are very similar to each other irrespective of the differences in methods, which suggests compatibility between the methods and, in addition, reliability of the experimental results.

### Characteristics of non-CT porosimetric data

**Figure 3:**
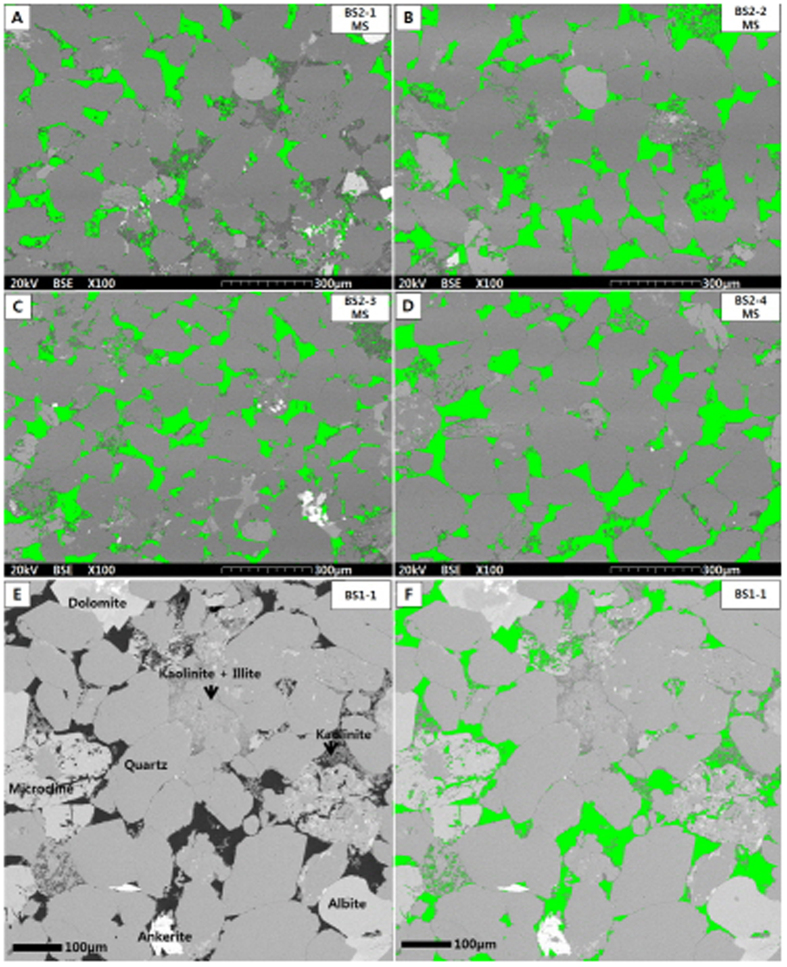
Petrographic image analysis and mineral identification on scanning electron microscopic images. Note relatively large intergrain pores in (**B**,**D**), whereas smaller ones in (**A**,**C**). Note also presence of micron to nano-scale intragrain pores within some grains or clay mineral aggregates filling the original pore space in (**E**,**F**).

Analyses of the samples using scanning electron microscopy (SEM), energy dispersive spectroscopy (EDS) and X-ray diffraction (XRD) methods suggest that the zigzag trend in porosity variations is closely related to the textural and mineralogical characteristics of the samples. Figure 3 shows an example of scanning electron microscopy and petrographic image analysis (SEM_PIA), which indicates the dominance of micron-scale interparticle (intergrain) pores in the samples with high porosities (group numbers BS1-2, BS1-4, BS2-2 and BS2-4)(Fig. 3B,D), and the subordinating occurrence of smaller intergrain pores but of the same micron scale in the samples with low porosities (group numbers BS1-1, BS1-3, BS2-1 and BS2-3)(Fig. 3A,C). Furthermore, samples with higher porosity are “cleaner” (i.e., they contain less detrital matrix), and have higher contents of quartz framework grains and lower contents of minor minerals such as clay minerals, feldspars and dolomites (Fig. 3E,F) (See also supplementary Fig. S4 and Table S1). The reverse is also true, i.e., samples with lower porosity have more detrital matrix, lower contents of quartz framework grains, and higher contents of other minor minerals. Some portions of intergrain pores are filled with clay mineral aggregates which also have intragrain pores of micron to nano scale, and such tiny intragrain pores are also found within some plagioclase and dolomite grains (Fig. 3E,F). The less clean samples are generally low in porosities irrespective of tiny intragrain pores within the grains (Figs 1 and 2). These observations suggest that the higher porosities of the Berea Sandstone samples are mainly due to a large proportion of intergrain pores to total rock volume, whereas the lower porosities are most likely caused by partial filling of pore space with clay mineral aggregates (cements). In other words, even though the minor minerals contain abundant intragrain pores, their presence is associated with clay mineral cementation and, as such, decreased porosity in the samples analyzed.

**Figure 4:**
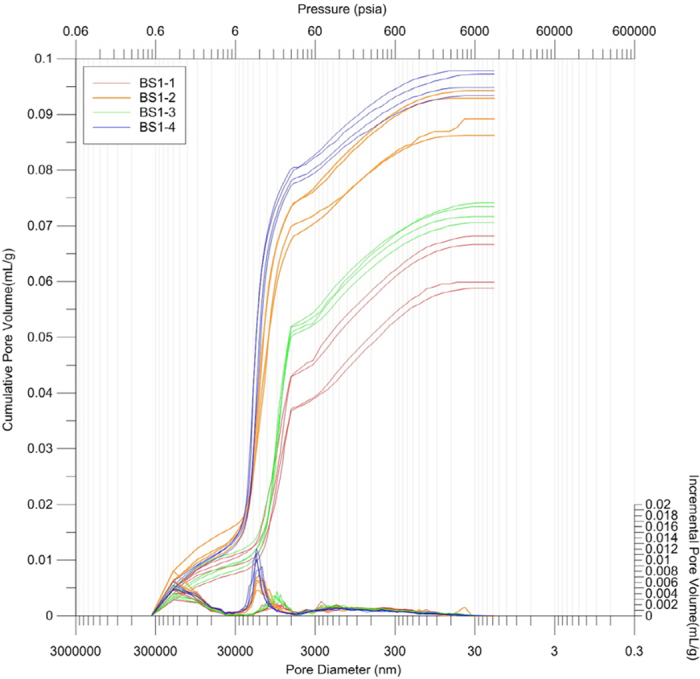
Results of the mercury intrusion (MI) method for the porosity measurement. Individual incremental volumes and cumulative volume of mercury are shown by curves plotted along with increasing pressure on the X axis, i.e., decreasing size of mercury-intruded pores. Note modal distribution of pores in three size intervals of 10^2^–10^1^ microns, 10^1^–10^0^ microns and 10^0^–10^−2^ microns, and higher proportion of the middle-sized pores (10^1^–10^0^ microns) in samples of higher porosities such as BC1-2 and BC1-4 (See also Fig. 1).

The MI method, normally operated under pressures of up to 60,000 psi, ensures an accurate measurement of porosity because the high pressures make mercury infiltrate into pores that are very small, down to a few nanometers in diameter (e.g. refs 4,14,15). As shown in Fig. 1, the MI method shows the clearest zigzag trend of porosities, compared to other multiple porosimetric methods applied concurrently to the first sample set. This method also provided excellent reproducibility, in that repeated measurements yielded the narrowest range of porosities, with variations amounting only 2–3% within each group of the sample set (See also supplementary Table S2). Hence the results of the MI method are taken as the norm for further evaluation of other porosimetric data. Moreover, the record of successive pressure increments in this method enables us to appreciate pore size variation within the samples^15^. In this study, the statistic treatment of pressure increment records evinces the presence of various-sized pores as observed on SEM images (Fig. 4). The records characteristically show modal distributions of pores in three separate size intervals of 10^2^–10^1^ microns, 10^1^–10^0^ microns and 10^0^–10^−2^ microns, with higher proportions of the middle-sized pores (10^1^–10^0^ microns) in samples of higher porosities such as BC1-2 and BC1-4 (Fig. 4).

In case of the HI method, the zigzagging trend of porosities along with arranged sample group numbers is also conspicuous but the porosities are significantly higher and have a greater variation than those obtained by the MI method (Fig. 1; See also supplementary Table S2). The difference between HI and MI porosities exceeds 5% on average throughout all the sample groups, and the deviation of HI porosities reaches approximately 5% within each group. Both MI and HI methods are known to measure various grades of pore sizes ranging from hundreds microns down to a few nanometers^22^. Here the reason for the significant gap between MI and HI porosities is unclear. It may be caused by either the presence of very small pore throats permitting entrance only of smaller helium molecules^17^, untreated conformance and compressibility corrections in MI method^23^, or the inherent weakness of the HI method adopted in this study. The HI method brought the bulk volume (or the bulk density) data from the MI method using a different sample container in a different equipment (Refer to the method). None the less it is noteworthy that both MI and HI porosities show the zigzag trends very similar to each other (Fig. 1), suggesting that both methods probably capture information from all pore sizes, from microns down to nanometers, and incorporate them into porosity calculation.

Figure 2 also displays the results of the SEM_PIA method applied to both the two sample sets, where the measured porosities are plotted along with arranged sample group numbers from BS1-1 to BS1-4 or from BS2-1 to BS2-4. The different squares in Fig. 2 represent different polished sections belonging to either the first or the second sample set, and the individual one expresses the statistically treated average of plural SEM_PIA results derived repeatedly from the different quarters of the surface of an individual polished section (See also supplementary Figs S3 and S5 and Table S3). The variation in porosity values obtained by this method is greater than those of other methods (Fig. 2), suggesting that the SEM_PIA method is disadvantageous because inaccuracy is introduced when the volume is extrapolated from the 2D slice image. Although rock matrix, macropores and micropores are clearly discernible on individual image slices in this method (Fig. 3), any single image slice is not likely sufficient for exacting the porosity of a rock sample. Instead, a statistical treatment of porosities from a number of plural image slices makes porosity values converge towards a value that is as accurate as those derived from other methods (See supplementary Fig. S5 and Table S3). Hence it seems that an accurate quantification of 3D pores requires data assimilation and statistic treatment of narrow-ranged serial 2D slices as many as possible, which is surely as important as the endeavors for exacting segmentation of the features over an improved high resolution image of a single 2D slice (e.g. refs 7,24,25). Here a significant insight can be drawn, i.e., the CT method treating a great number of successive image slices thus being three dimensional in practice is more appropriate for an accurate measurement of rock porosity.

### Characteristics of CT porosimetric data

**Figure 5:**
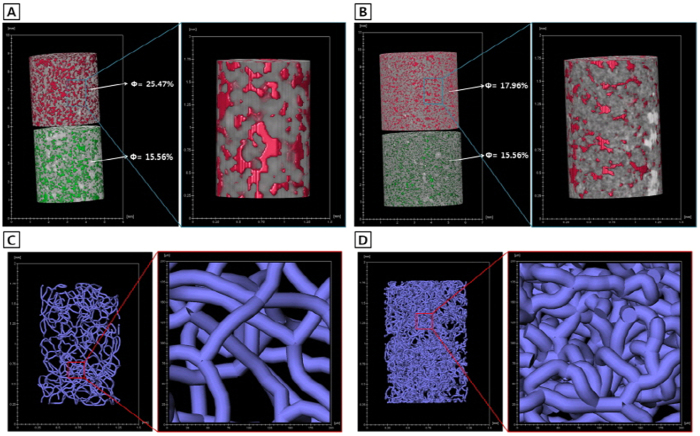
Examples of porosity measurement and pore visualization using the reference-sample-guided computed tomography of either low-resolution control or high-resolution control for the same Berea Sandstone sample. Note probable simplification of the original pore networking under low-resolution controls, evinced by large pore segmentations (**A**) and loose centerlines of filamentous pores (**C**). Note also a proper pore networking under high-resolution controls showing sufficiently small pore segmentations (**B**) and compact centerlines of filamentous pores (**D**).

Figure 1 also shows porosities of the CT_LR method, plotted together with the BC and MI porosities. Here, the MI porosities, showing the best performance results, can be used as a norm for the evaluation of the CT porosities (See also supplementary Table S4). Following the method of Jin *et al*.^21^, we have derived, first of all, an appropriate gray level from the reference sample (RS) using the interactive thresholding process which makes the porosity be coincident with the MI porosity of the same RS (Fig. 5)(See also supplementary Fig. S3). Then the gray level has been applied to the measurement sample (MS) for the calculation of porosity (Refer also to the method). As a result, the CT_LR porosities of the MS also show the trend zigzagging with the arranged sample group numbers, similar to the trends of the MI and HI porosities. An in-depth inspection, however, indicates that the CT_LR porosities are on occasion over- or under-estimated, depending on how the MS is paired with the RS during CT scanning. As shown in Fig. 1, the pairings of a less porous MS (e.g., sample group nos. BS1-1 and BS1-3) with a more porous RS (e.g., sample group nos. BS1-2 and BS1-4) yield CT_LR porosities of the MS that are lower than the MI porosities of practically the same sample (See also supplementary Table S4). The reverse is also true, i.e., the pairings of a more porous MS with a less porous RS show CT_LR porosities higher than the relevant MI porosities. On the contrary, the pairings of MS and RS with porosities of a similar grade show the results that appear to somewhat contradict each other. In case of the pairings of a less porous MS with a less porous RS, CT_LR porosities of the MS are close to the relevant MI porosities, whereas the pairings of a more porous MS with a more porous RS result in CT_LR porosities of the MS that are considerably lower or higher than the relevant MI porosities (See also supplementary Table S4 for detailed explanation).

Contrastingly, Fig. 2 shows porosities of the CT_HR method of which experimental steps are identical to those of the CT_LR method but were applied to the second sample set under significantly improved resolution control during CT scanning. As shown in Fig. 2, the relationship between CT_HR and MI porosities are rather simple and straightforward, compared to those of CT_LR and MI porosities of the first sample set (cf. Figs 1 and 2). When plotted along with the arranged sample group numbers from BS2-1 to BS2-4, the CT_HR porosities show a zigzag trend almost exactly the same as that of the MI porosities (Fig. 2) (See also supplementary Table S5). Moreover, the values of the CT_HR porosities are fairly coincident with those of the MI porosities, both having similarly narrow-ranged deviations. The poor agreement between CT_LR and CT_HR methods versus the good agreement between CT_HR and MI methods suggests that the resolution of the CT scanning is crucial to predicting porosity accurately using the CT method.

In Fig. 5, we can appreciate the possible cause of the inaccurate estimation of porosities by low resolution CT scanning. The pore network of the MS appears much rougher in the low resolution tomograms (Fig. 5A), relative to the high resolution tomograms (Fig. 5B) showing a marked coincidence between the MI and CT_HR porosities (Fig. 2). Hence we can infer that some pores in the MS would be overlooked or overweighed at low resolution. These observations further suggest that the reference-sample-guided CT method could be a good means for porosity measurement provided the CT system satisfies the necessary resolution.

Pores in reservoir rocks commonly exist over the full range of the scale in size from larger than millimeters to smaller than nanometers. Hence, different observational equipments, individually having a limited range of views and resolution, have been employed, and resultant multi-scale, multi-resolution data have to be integrated^20^,^26^. The reference-sample-guided CT method can be a convenient way to estimate pore characteristics to some extent of the pore size smaller than the original coverage of the CT equipment, i.e., the CT resolution of the prior set up. The resolution is not necessary to be much better than a few microns for the porosity measurement of medium to very fine sandstones, although the rocks may include some mud aggregates and micron to nano-scale intraparticle pores inside. Higher resolution controls are, however, necessary for the measurement of a finer-grained rock. Considering the additional merit of the CT method visualizing the pore structure in 3D, the reference-sample-guided CT method is very competitive with or even more effective than the other porosimetric methods when it attains the minimum requirement of the resolution control for a specified rock type.

## Discussion

Our experimental results show a good correlativeness of porosities by the multiple methods such as mercury intrusion (MI), helium injection (HI), petrographic image analysis on scanning electron microscopic images (SEM_PIA) and computed tomography of either low resolution (CT_LR) or high-resolution (CT_HR). Porosities from the MI method are highly reliable, showing a small deviation within each sample group. The CT_HR method also yields a high-quality data matched well with that of the MI porosity. The reference-sample-guided computed-tomographic method is appropriate for the porosity measurement of reservoir rocks if it is under an adequate resolution control. An inadequate low resolution in the CT method would result in over- or under-estimation in porosity measurements thus further researches are necessary to determine optimum resolutions for specified rock types.

## Methods

### Sample preparation

Berea Sandstone is one of the oil and gas reservoirs, which was deposited under subaerial to shallow subaqueous environments during the Lower Carboniferous (Mississippian) period (e.g. Pepper *et al*.^27^). Using four medium to fine-grained Berea Sandstone samples (short cores) different from each other, we prepared two sample sets, designated BS1-* and BS2-*, to apply multiple porosimetric methods systematically (See supplementary Figs S1, S2 and S3). In order to make the first sample set (BS1-*), we performed close-spaced drillings, iterated six times on top of each core, thus six tiny core plugs were retrieved from each core, which are individually ca. 5 mm in diameter and 2–3 cm long. Hence, the first sample set consists of four groups designated BS1-1, BS1-2, BS1-3 and BS1-4, each group comprising six tiny core plugs representing the corresponding Berea Sandstone core (See also supplementary Fig. S2). Among the six tiny core plugs of each group, four were used for the course of experiments from helium gas injection to mercury intrusion. Of the rest two, one was used for the experiment of CT in three dimensions (3D), whereas the other was for SEM_PIA in two dimensions (2D). In the CT experiment, a core plug of a group was paired with another core plug of a different group and both were x-ray scanned concurrently to adopt the method of the reference-sample-guided computed-tomographic porosimetry as introduced in our earlier works (e.g. Jin *et al*.^21^). The first-chosen core plug was for the usage as a reference, i.e., reference sample (RS), whereas the second-chosen core plug was for porosity measurement itself, i.e., measurement sample (MS).

In order to make the second sample set (BS2-*), we cut and discarded the uppermost part of the original core used for extracting the first sample set because it was ruined by the first-time close-spaced drilling (See also supplementary Figs S2 and S3). On the refreshed top of each core, we repeated close-spaced drillings twice to take two tiny core plugs similar in size to those of the first sample set. Hence this second sample set also consists of four groups designated BS2-1, BS2-2, BS2-3 and BS2-4 but here each group comprises only two tiny core plugs. This sample set was mainly for the re-application of computed tomography but under significantly improved resolution control than that of the first sample set. We prepared a full set of two tiny (mm-scale) core plugs from each group, i.e., one only for the usage as reference (RS) and the other only for measurement (MS) (See also supplementary Fig. S3). The pair of reference and measurement samples (RS and MS) was also X-ray scanned concurrently as is the case of the first sample set. An exact comparison between CT and SEM_PIA results was also made by means of using the same core plug for the two methods (See also supplementary Fig. S3).

### Computed-tomographic porosimetry

We utilized a computed tomography (CT) machine in the Korea Institute of Geoscience and Mineral Resources (KIGAM). The machine is equipped with two switchable X-ray tubes of 225 kV and 160 kV, one flat panel detector with 2048 * 2048 pixels (optional binning possible) and one rotary stage of 36 arcsec (~0.001°) resolution. In order to adopt Jin *et al*. (2013)’s method using a reference sample in computed-tomographic porosity measurement, we paired measurement sample (MS) and reference sample (RS) during CT scanning, putting the MS at top and the RS at bottom on the rotary sample stage (See also supplementary Fig. S3). For the experiment of the first sample set (groups from BS1-1 to BS1-4), we scanned the paired MS and RS with application of X-ray tube 225 kV (micro-focus) and detector pixel binning 1024*1024 (See also supplementary Table S6). In contrast, X-ray tube 160 kV (micro-focus) and detector pixel binning 2048*2048 were applied to the second sample set (groups from BS2-1 to BS2-4) in which MS and RS were paired in the same manner (See also supplementary Table S6). This type of sample deployment enabled us to apply the same scanning conditions to both MS and RS, and the configuration and input parameters made us ensure 15 microns resolution of the tomogram voxels for the first sample set and 5 microns resolution of those for the second sample set (See also supplementary Table S6).

We exploited two softwares for the processing of the X-ray scanned data: VG Studio MAX v.2.1 for tomogram production throughout 3D volume rendering; Avizo Fire v.8.1.0 for further data treatment including porosity calculation. When using VG Studio MAX v.2.1, data processing was made over a data set consisting of both MS and RS in order to keep the same conditions on both samples. Based on input parameters, we performed offset and tilt correction, and then set the region of interest (ROI) and the extent of beam hardening correction for both MS and RS. In the next step, we performed volume rendering thus finally acquired 3D tomogram and object property information including dimension (size), xyz resolution, voxel counts and total volume.

Using Avizo Fire v.8.1.0, we re-processed the raw file of the tomogram sent from the VG Studio MAX v.2.1, referring to the object property information of the previous step. Following Jin *et al*.^21^’s concept using a reference sample in computed-tomographic porosimetry, the reprocessing was performed first over the RS. In practice, we took a volumetric crop from the RS, taking only the central part of the original volume to remove any possible erroneous effect at the margin. Then we applied a volume-editing process to the crop to specify a sub-volume of calculation with original voxel values (a full range of original gray levels) and a fixed volumetric extent. Then we performed the interactive thresholding process by means of applying a range of gray levels repeatedly to the crop until the calculated porosity is best matched with the given (known) porosity of the RS, i.e, the MI porosity in this study. Referring to the given voxel positions and the range of gray levels, we could draw out CT porosity (*ϕ*_*CT*_) for the extracted sub-volume, using the equation (1).


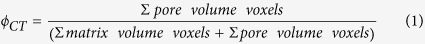


We iterated this process until both CT and MI porosities of the RS became almost the same as each other, i.e., those are within the difference of 0.5%. When we obtained the most appropriate gray-level range for the RS, we applied it to the MS to calculate the porosity using the same equation as (1).

### SEM observation and mineral identification

In the first sample set, a core plug different from those for the CT and MI methods but very close in extraction position was picked up and manufactured into a polished section for the SEM observation. Contrastingly, in the second sample set, the two core plugs in each sample group were first used as either MS or RS, and then exploited again for polished sections. We impregnated the core plugs using Epo Thin^TM^ epoxy resin (20-8140-128) and hardener (20-8142-064), and polished sections were made by an automatic grinder/polisher of Echomet 250 & Automat 250 (Buehler, model 49-7250). The alumina polishing suspension (0.05 μm) of MASTERPREP was used during the work. The polished surface was coated by platinum 2 nm thick. We used SEMs of JSM-7610F (JEOL co.) and SNE-3000M (SEC co.) for the recognition of rock matrix, pores and some particular minerals. We acquired 2D BSE image slices of 100 diameters in normal cases and 500–1000 diameters in an extreme case, both enabling us to discriminate matrix, macropores (e.g., intergrain pores) and even micropores (e.g., intragrain pores). For the quantitative analysis, we normally used the petrographic image slice of 100 diameters and 1280 * 1024 pixels which was obtained under electron acceleration voltage of 10 kV. We utilized a self-made program to calculate porosity based on an interactive thresholding of gray levels on the image.

In order to find any relationship between matrix composition and pore characteristics, we examined mineralogy of the matrix using SEM/EDS and XRD instruments. The EDS tool (model X-Max^N^ 50, Oxford co.), attached at the SEM, enabled us to recognize high-proportioned matrix minerals of different gray levels. The XRD tool (D8 ADVANCE with Bragg-Brentano geometry, Bruker co.) and appended softwares (DIFFRAC.EVA and Bruker TOPAS) were utilized to quantify the matrix mineralogy in detail.

### Petrographic image analysis and porosity determination

Over the polished section of each sample, we randomly collected 100-diameter SEM image slices, and performed porosity determination on each collected slice using a self-made image analysis program. The program functions similarly to other image analysis programs of commercial base such that we can segment pores and non-pore area (matrix) on the black and white image using a proper gray-level range determined by an interactive thresholding process. Repeated comparisons of larger-diameters (e.g, 500X or 1000X) raw-SEM image and program-treated segmented image at the same position enabled us to determine the most proper gray level for the segmentation and to calculate the SEM_PIA porosity (*ϕ*_*SEM_PIA*_) using the equation (2).


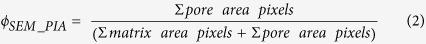


### Helium injection

The four core plugs in each group of the first set were used for helium injection to examine grain volume of the sample. Prior to the helium injection, we measured weight of the individual plug using the mettler of TOLEDO model AB204-S having readability of 0.1 mg and repeatability of 0.1 mg. The helium injection process was performed by the pycnometer of model AccuPyc^TM^II 1330 (Micromeritics co.) which can transform the measured matrix volume into the matrix density using the separately measured sample weight.

### Mercury intrusion

The core plugs, which were used for measurement of matrix density using the pycnometer, were also used for the application of the mercury intrusion (MI) method. Each core plug was weighed again prior to the MI method application. In order to make mercury intrusion into the pores of the core plug, we used the equipment of AutoPore IV 9520 (Micromeritics co.) which has two separate pressure ports for the measurement of different pressure intervals: the lower pressure port for 0.10–30 psia and the higher pressure port for 30–60,000 psia. This equipment can provide us with a cumulative record of mercury intrusion volume increasing with ambient pressure rise from 0.10 psia up to 60,000 psia, i.e., a continuous record of mercury increments to intrude the pores decreasing in size from 360 μm down to 0.003 μm, inversely to the increasing pressure. Hence the maximum intrusion volume can be read on the cumulative curve, i.e., it is indicated by the point on the curve with no more increase in mercury intrusion volume even under further increase in pressure.

### Porosity calculations in MI and HI methods

We followed the guideline of the equipment manual for the calculation of the Mercury Intrusion (MI) porosity. First of all, we derived the bulk density of each core plug from the experiment, using the measured values (sample volume and weight) and the equation (3)^23^.


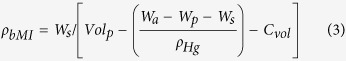


where *ρ*_*bMI*_ is the bulk density from MI method in g/cm^3^, *W*_*S*_ is the weight of the sample in g, *Vol*_*p*_ is the volume of the penetrometer in cm^3^, *W*_*a*_ is the weight of the apparatus including sample and mercury in g, *W*_*p*_ is the weight of the empty penetrometer in g, *ρ*_*Hg*_ is mercury density in g/cm^3^, and *C*_*vol*_ is the conformance volume in cm^3^.

Based on the derived bulk density, we also calculated porosity of each core plug, using the following equation (4)^23^.


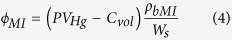


where *ϕ*_*MI*_ is the porosity from the MI method in volume fraction, *PV*_*Hg*_ is the pore volume of mercury injected at 60,000 psia in cm^3^.

We used the pycnometer for the measurement of Helium Injection (HI) porosity, and adopted the bulk density from the mercury intrusion, following the recommendation of API RP40^3^. As mentioned earlier, the HI method preceded the MI method in our experimetal procedure. The equation (5) for the HI porosity calculation is as follows.


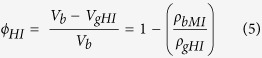


where *ϕ*_*HI*_ is the porosity from the helium porosimeter in volume fraction, *ρ*_*bMI*_ is the bulk density from MI method in g/cm^3^, *ρ*_*gHI*_ is the grain density from the helium porosimeter in g/cm^3^, *V*_*b*_ is the bulk volume, which can be measured by calipers or mercury immersion^3^. Here we used the second equation to determine the HI porosity in which the bulk density was adopted from the MI method.

## Additional Information

**How to cite this article**: Jin, J. H. *et al*. Correlative multiple porosimetries for reservoir sandstones with adoption of a new reference-sample-guided computed-tomographic method. *Sci. Rep.* **6**, 30250; doi: 10.1038/srep30250 (2016).

## Supplementary Material

Supplementary Information
