# Supplementary material for: Correlative multiple porosimetries for reservoir sandstones with adoption of a new reference-sample-guided computed-tomographic method
Source: Sci Rep. 2016 Jul 22;6:30250. doi: 10.1038/srep30250 (PMC4957228; doi:10.1038/srep30250)
Supplement: Supplementary Information [file srep30250-s1.pdf]

Supplementary Materials

Correlative multiple porosimetries for reservoir sandstones with adoption of a new reference-sample-guided computed-tomographic method

Jae Hwa Jin*, Junho Kim, Jeong-Yil Lee & Young Min Oh

Korea Institute of Geoscience and Mineral Resources, 124 Gwahak-ro, Yuseong-gu, Daejeon, 34132 Korea

***jhin@kigam.re.kr**

Supplementary Figure S1

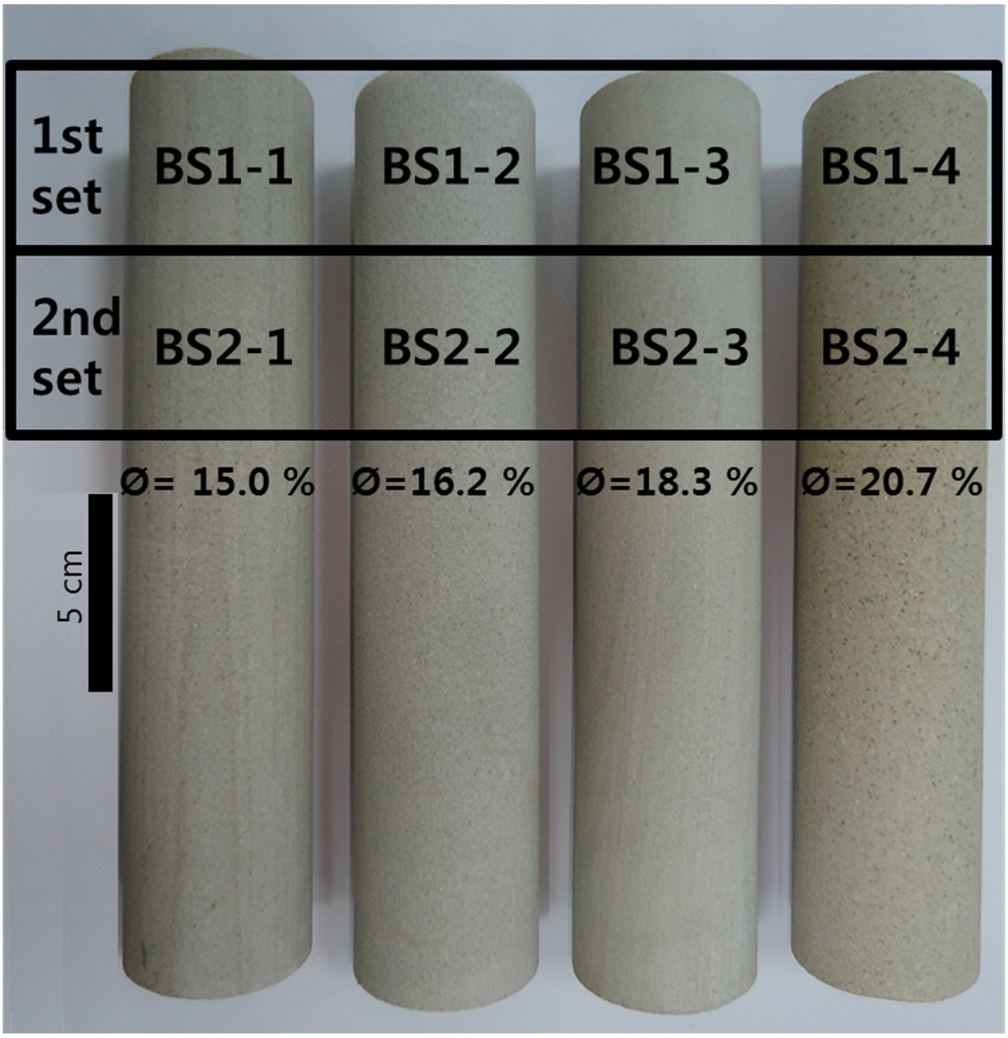

A total of four Berea Sandstone cores were used for the experiment of this study. We made two different sample sets (1st set and 2nd set), each comprising four core plugs (segments). Each core plug in the first sample set represents an individual sample group comprising tiny (mm-scale) core plugs extracted from it and has a twin (practically the same sample) in the second sample set. Because the space of the individual core plug top was limitedly available, tiny (mm-scale) core plugs were insufficiently extracted from the core plugs of the first sample set thus additional tiny core plugs were supplemented from the second set to apply various porosimetric methods. See the following supplementary Figs. S2 and S3 for further information of the subsamples. The porosities (ϕ) described on cores were those notified from the company selling the samples, designated here as BC porosities.

Supplementary Figure S2

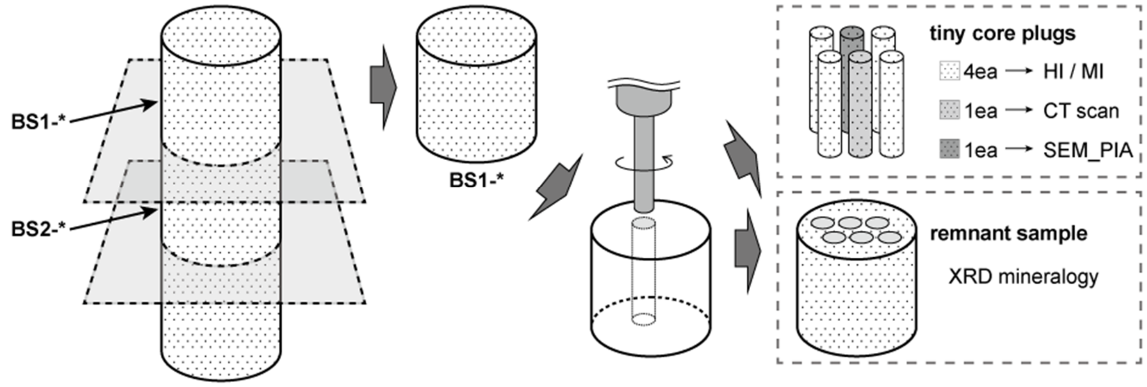

Two successive core plugs (segments) were obtained from an original Berea Sandstone core, which were designated BS1-* and BS2-* respectively. A total of six mm-scale cores were extracted from the first plug (BS1-*), and then four among the six were used for the porosimetric experiment of helium injection method followed by mercury intrusion method. The rest two were for the porosimetries of CT and SEM_PIA methods. The top of the first plug was almost entirely ruined by the dense subsampling thus we had to secure the second plug (BS2-*) to extract additional mm-scale cores for further experiment of different items.

Supplementary Figure S3

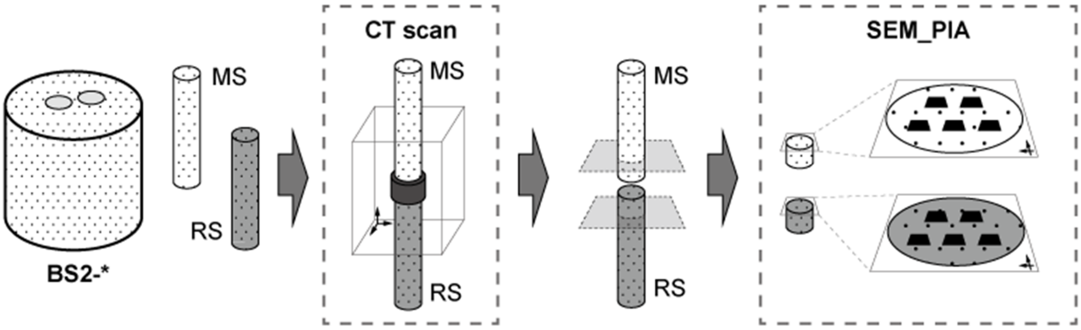

The second core plug from an individual Berea Sandstone core, i.e., BS2-*, was used to extract two mm-scale core plugs, both for the porosity measurement using computed tomography (CT) under significantly improved resolution control, compared to the CT experiment of low resolution in the first sample set. Here one plug was used only for the sample of porosity measurement (measurement sample, MS), whereas the other was used only for the sample of reference (reference sample, RS). Adopting the concept of the reference-sample-guided computed tomography (Jin et al., 2013), a measurement sample and a reference sample were always paired to be scanned concurrently using the CT system (the same experimental procedure as it was in the first sample set). After the CT scanning, the mm-scale core plugs were manufactured into polished section for the application of the SEM_PIA method.

Jin, J. H., Kim, J. H. & Kim, M. J. System for measuring sample pore using computed tomography and standard sample and method thereof. Patent US8542793 B1, PCT/KR2011/007269. (2013).

Supplementary Figure S4

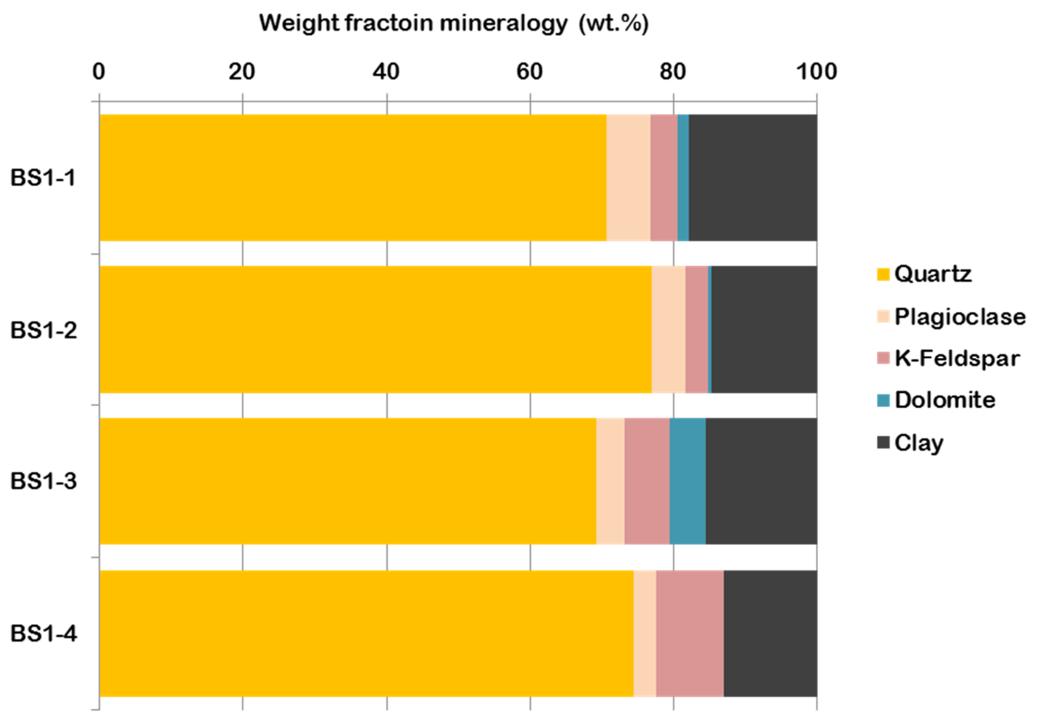

Mineralogic composition was examined using the XRD equipment D8 ADVANCE and accompanied softwares DIFFRAC.EVA and Bruker TOPAS. Quartz is predominant throughout all the samples. Quartz contents are low in sample group numbers BS1-1 and BS1-3 and high in BS1-2 and BS1-4, similarly with the trend of porosities (See also the main Figs. 1 and 2 for porosities). In case of the sum of other minerals, the reverse trend is true.

Supplementary Figure S5

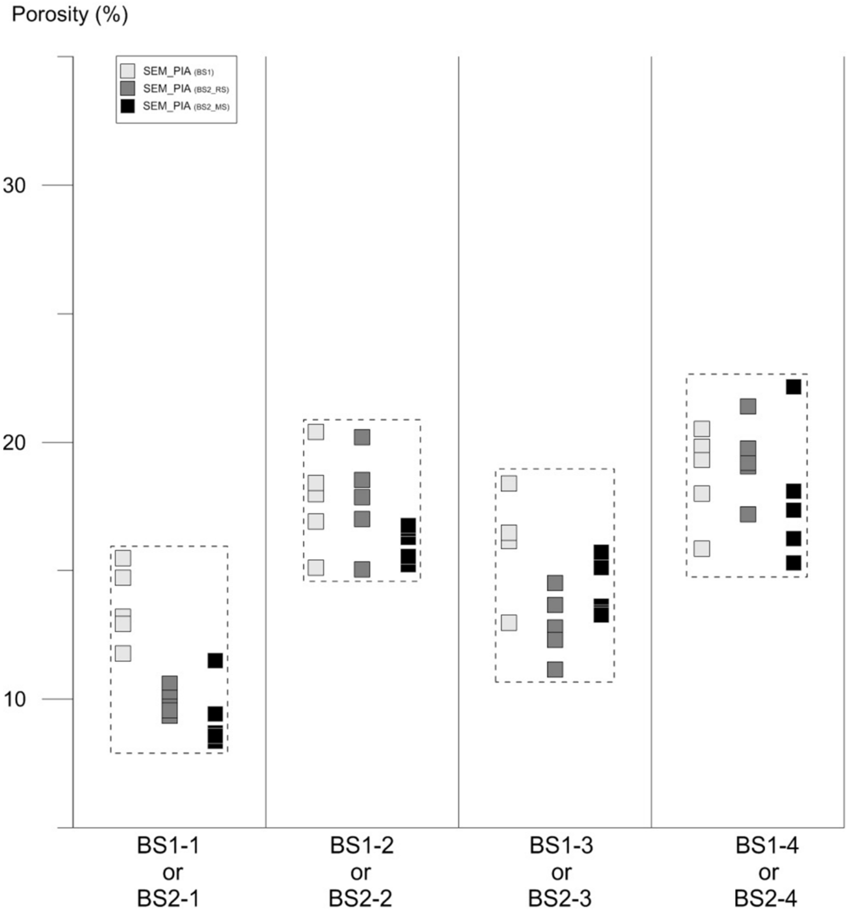

Results of petrographic image analysis on scanning electron microscopic images (SEM_PIA) are shown. Note sample group numbers belonging to either the first sample set (light gray squares) or the second one (dark gray and black squares). The same gray-tone squares in each sample group represent a single polished section and an individual square corresponds to a different quarter of the polished section surface. Note also a large box with the dashed line which envelops all the squares in each sample group. The clusters of squares indicate a zigzagging variation of porosities along with the arranged sample group numbers, similarly with the trend of porosities measured by other porosimetric methods.

Supplementary Table S1

Group	Quartz	K-Feldspar	Plagioclase	Dolomite	Clay	Grain Density [g/cc]
BS1-1	70.66	3.78	6.06	1.63	17.87	2.66
BS1-2	76.92	3.05	4.77	0.54	14.72	2.66
BS1-3	69.15	6.22	4.05	5.03	15.56	2.67
BS1-4	74.39	9.45	3.17	0.00	12.99	2.65

Results of mineralogic analyses using the XRD equipment D8 ADVANCE are shown. Mineral contents are in weight percentage (wt. %). Note that quartz contents are low in sample group numbers BS1-1 and BS1-3 and high in BS1-2 and BS1-4.

Supplementary Table S2

Group	mercury intrusion (MI) porosity (%)					helium Injection (HI) porosity (%)				
	porosity 1 (sample 1)	porosity 2 (sample 2)	porosity 3 (sample 3)	Porosity 4 (sample 4)	Avg. of Porosity	porosity 1 (sample 1)	porosity 2 (sample 2)	porosity 3 (sample 3)	porosity 4 (sample 4)	Avg. of Porosity
BS1-1	12.85	13.96	12.91	14.90	13.66	21.58	23.22	19.35	18.05	20.55
BS1-2	17.74	18.43	18.92	19.35	18.61	24.72	24.74	25.59	22.64	24.42
BS1-3	15.52	15.92	15.09	16.01	15.63	20.63	19.84	21.62	19.29	20.34
BS1-4	19.83	19.51	19.05	20.20	19.65	25.07	24.28	24.46	20.81	23.65

Results of porosity measurements using mercury intrusion (MI) and helium injection (HI) methods are shown. Both methods were applied together in a series to each sample using different equipments, i.e., first helium injection by the pycnometer AccuPyc^{TM}II 1330 and second mercury intrusion by AutoPore IV 9520. In calculation of HI porosities, the data of bulk volume (or bulk density) were brought from the records of the MI method.

Supplementary Table S3

Sample sets	Group	SEM_PIA Porosity	Avg. of SEM_PIA Porosity	Sample sets	Group	SEM_PIA Porosity	Avg. of SEM_PIA Porosity	Sample sets	Group	SEM_PIA Porosity	Avg. of SEM_PIA Porosity
BS1	BS1-1	11.78	13.62	BS2_RS	BS2-1	9.36	9.85	BS2_MS	BS2-1	8.69	9.31
		13.20				10.59				8.36	
		14.71				10.04				8.56	
		15.48				9.69				9.43	
		12.92				9.55				11.49	
	BS1-2	15.11	17.76		BS2-2	20.21	17.72		BS2-2	16.29	16.09
		20.40				17.00				16.62	
		17.97				17.85				15.23	
		16.91				15.05				15.55	
		18.41				18.51				16.74	
	BS1-3	12.97	16.07		BS2-3	11.15	12.88		BS2-3	13.61	14.21
		16.38				13.66				13.35	
		16.15				14.50				15.71	
		18.39				12.79				13.26	
		16.46				12.29				15.10	
	BS1-4	17.99	18.70		BS2-4	19.05	19.31		BS2-4	18.07	17.82
		15.85				19.75				16.24	
		19.83				19.18				22.15	
		20.52				17.19				15.30	
		19.30				21.40				17.35	

Results of porosity measurements using the method of petrographic image analysis on scanning electron microscopic images (SEM_PIA) are shown. In every polished section designated as the individual group number, the SEM_PIA analyses were iterated five times, and the outputs were averaged (See also supplementary Fig. S5). The average values are plotted in the main Fig. 2.

Supplementary Table S4

MS \ RS	BS1-1 (13.7)	BS1-2 (18.6)	BS1-3 (15.6)	BS1-4 (19.6)
BS1-1	·	9.33	13.65	8.91
BS1-2	25.39	·	25.47	22.43
BS1-3	13.75	10.43	·	9.82
BS1-4	27.67	16.32	27.20	·

* The value in parenthesis of individual RS numbers represents the porosity value adopted from the MI porosity of practically the same sample and assigned to the RS in order to derive the measured porosity from individual RSs.

Results of porosity measurements are shown, applying the method of computed tomography of low resolution, i.e, the CT_LR method, to the first sample set. A reference sample (RS) and a measurement sample (MS) were always paired during the CT scanning. When a sample of a specified group was chosen as the RS, we also chose the other one of the remaining sample groups as the MS and stacked it on the RS to scan them concurrently using the CT system. This type of pairing of RS and MS was repeated until a full round of the experiment was made. Note that no data exists where sample group numbers of RS and MS are the same because only one sample was used among six samples of each group in the first sample set. The pairings of a less porous MS with a more porous RS (e.g., pairing of MS BS1-1 and RS BS1-2 or RS BS 1-4) yield CT_LR porosities of the MS lower than the MI porosities of practically the same sample. The reverse is true, i.e., the pairings of a more porous MS with a less porous RS (e.g., pairing of MS BS1-2 and RS BS1-1 or RS BS 1-3) show CT_LR porosities higher than the relevant MI porosities. On the contrary, the pairings of MS and RS with MI porosities of a similar grade show results somewhat contradictive with each other. In case of the pairings of a less porous MS with a less porous RS (e.g., pairing of MS BS1-1 and RS BS1-3 or MS BS1-3 and RS BS1-1), CT_LR porosities of the MS are close to the relevant MI porosities, whereas the pairings of a more porous MS with a more porous RS (e.g., pairing of MS BS1-2 and RS BS1-4 or MS BS1-4 and RS BS1-2) result in CT_LR porosities of the MS that are considerably higher or lower than the relevant MI porosities.

Supplementary Table S5

RS / MS	BS2-1 (13.7)	BS2-2 (18.6)	BS2-3 (15.6)	BS2-4 (19.6)
BS2-1	14.36	13.84	11.65	14.68
BS2-2	18.53	19.16	17.96	19.74
BS2-3	15.83	15.88	15.47	15.51
BS2-4	19.20	18.87	18.32	19.98

* The value in parenthesis of individual RS numbers represents the porosity value adopted from the MI porosity of practically the same sample and assigned to the RS in order to derive the measured porosity from individual RSs.

Results of porosity measurements are shown, applying the method of computed tomography of high resolution, i.e, the CT_HR method, to the second sample set. As in the CT_LR method, a reference sample (RS) and a measurement sample (MS) were always paired during the CT scanning (See supplementary Fig. S3 for the reason). Here two samples (mm-scale tiny core) were brought from each group, one only for the usage as the RS and the other only as the MS. When one RS was chosen, we also chose one MS, and stacked both vertically to scan them concurrently using the CT system. This process of sample selection and CT scanning was repeated until a full round of the experiment was made. Note that data exists where the sample group numbers of MS and RS are the same because both RS and MS samples were separately collected in the second sample set.

Supplementary Table S6

Parameters / Sample set no. (original x-ray tube voltage)	Detector Binning mode	X-ray Tube Voltage (kV) / Current (μA)	View number	Averaging number	Readout time (ms)	Detector size (mm)	Source to object (mm)	Object to detector (mm)
BS1-* (225kV for CT_LR)	1024 * 1024	140/80	1440	16	100	409.6 *409.6	31.708	825.382
BS2-* (160kV for CT_HR)	2048 * 2048	90/70	2880	16	100	409.6 *409.6	29.85	1082.448

This table shows two different configurations of the CT components applied differently to either the first or the second sample set. Naturally input parameters are also different from each other. This prior set up of CT components and input parameters allowed us to get image resolution of 15 microns for the first sample set (BS1-*) and 5 microns for the second sample set (BS2-*)

Supplementary Information S1

Additional information of personal e-mail communications May 2016 and contents summary

darleneo@clevelandquarries.com (The company selling Berea sandstone samples)

Raw blocks vary in size. They can be as large as 7-9 ft in length, 3-5 ft in width, and 2-4 ½' in height. Samples that are tested at Weatherford Labs are only 1 ½" dia x 2" long cut from these large blocks. The values we receive after Weatherford tests the samples are the n assigned to the whole block. That is why the cores cut from the blocks are sometimes q uite different from the values assigned, and why we say the values are for reference only, and not to be used for testing purposes.

Paul.Lincoln@weatherfordlabs.com (The laboratory testing Berea sandstone samples)

The grain volume of plug samples is measured by helium injection using the Boyle's Law method. Bulk volume determination is made by mercury immersion, Archimedes, or caliper measurements of length and diameter. Ambient porosity is then calculated using the formula:

Vb - Vsg=Vp

Vp / Vb x 100

Vb= Bulk volume

Vsg = Grain volume

Vp = Pore volume
